# Lower serum levels of Meteorin-like/Subfatin in patients with coronary artery disease and type 2 diabetes mellitus are negatively associated with insulin resistance and inflammatory cytokines

**DOI:** 10.1371/journal.pone.0204180

**Published:** 2018-09-13

**Authors:** Maryam Dadmanesh, Hassan Aghajani, Reza Fadaei, Khodayar Ghorban

**Affiliations:** 1 Department of Infectious Diseases, School of Medicine, Aja University of Medical Sciences, Tehran, IR, Iran; 2 Interventional Cardiology Department, Tehran Heart Center, Tehran University of Medical Sciences, Tehran, IR, Iran; 3 Department of Biochemistry, School of Medicine, Aja University of Medical Sciences, Tehran, IR, Iran; 4 Department of Immunology, School of Medicine, Aja University of Medical Sciences, Tehran, IR, Iran; University of Milan, ITALY

## Abstract

Meteorin-like (Metrnl) is a newly discovered adipokine with favorable effect on insulin sensitivity. Previous studies have reported lower levels of Metrnl in obese patients. However, there is conflicting data regarding its circulating levels in type 2 diabetes mellitus (T2DM) and there is no data in patients with coronary artery disease (CAD). The aim of the present study was to evaluate the Metrnl serum level in patients with T2DM and CAD, and also to evaluate the serum levels of Metrnl with serum levels of adiponectin, IL-6 and TNF-α in patients. This study was conducted on 66 patients with CAD, 63 T2DM patients and 41 controls. The serum levels of Metrnl, adiponectin, IL-6 and TNF-α were measured using ELISA techniques. The serum levels of Metrnl were found to be lower in CAD (75.18 ± 28.48 pg/mL) and T2DM patients (73.89 ± 33.60 pg/mL) compared to the control group (95.33 ± 32.56 pg/mL) (p < 0.005 and p<0.003, respectively). Additionally, adiponectin decreased in CAD and T2DM patients as compared to the control group, while IL-6 and TNF-α were higher in CAD and T2DM patients. Metrnl showed independent association with the risk of CAD and T2DM presence. Furthermore, Metrnl illustrated a negative correlation with IL-6 and TNF-α in both CAD patients and also with BMI, insulin resistance, IL-6 and TNF-α in T2DM patients. Metrnl showed an association with CAD and T2DM presence and with components of their pathogenesis such as inflammation and insulin resistance. These results suggested a possible interaction between Metrnl and the pathogenesis of CAD and T2DM, however more studies are needed to prove this concept.

## Introduction

The adipose tissue is an endocrine organ that secretes adipokines such as adiponectin, leptin, visfatin, resistin, vaspin, etc [1]. Adipokines play important roles in whole body glucose and lipid metabolism as well as in inflammation [1]. Additionally, obesity has a close relationship with cardiometabolic diseases such as type 2 diabetes mellitus (T2DM) and coronary artery disease (CAD) [2]. CAD is a leading cause of death worldwide, and atherosclerosis is the main underlying mechanism of CAD [3]. Atherosclerosis is a progressive inflammatory disease and results to the accumulation of lipids in the arterial cell wall [3–6]. T2DM is a chronic metabolic disease and insulin resistance and β-cell dysfunction are the main underlying mechanisms [7,8]. It has been reported that obesity is associated with chronic inflammation and dysregulation of adipokine secretion and could be a possible link between obesity and increased risk of T2DM and CAD [9].

Meteorin-like (Metrnl), also known as subfatin and cometin, is a newly discovered adipokine that is secreted by white adipose tissue and skeletal muscle [10–13]. It has been found that Metrnl increases during acute exposure to cold and induces thermogenesis associated genes in beige/brown adipose tissue [13]. Furthermore, Metrnl increases anti-inflammatory cytokines by promoting IL-4 expression [13]. Furthermore, several lines of evidence have shown the favorable effects of Metrnl on insulin sensitivity [12,14]. Metrnl increases insulin induced AKT phosphorylation, hence, it has been found that Metrnl deficiency leads to adipocyte insulin resistance. However, there are conflicting data regarding the circulating levels of Metrnl in T2DM [15,16]. In addition, there are no data regarding the circulating levels of Metrnl in patients with CAD. Moreover, it has been documented that T2DM and CAD patients suffer from increased expression of pro-inflammatory cytokines [17]. Therefore, the present study aimed to evaluate the serum levels of Metrnl in patients with T2DM and CAD, and its association with cardiometabolic variables and inflammatory cytokines.

## Materials and methods

### Subjects

This cross sectional study was conducted on 66 patients with CAD, 63 patients with T2DM and 41 healthy controls. The participants were recruited from Tehran Heart Center (Tehran, Iran) from April to December, 2017. CAD was diagnosed by a cardiologist according to the angiography results. Patients with > 70% stenosis in at least one coronary artery were diagnosed as CAD [18]. Furthermore, T2DM was diagnosed based on the criteria of the American Diabetes Association (ADA) [19]. Subjects without T2DM and CAD were included in the control group. Furthermore, patients with unstable angina, positive results of non-invasive test of heart function and any history of cardiovascular disease, including cerebrovascular, coronary artery and peripheral artery diseases and acute coronary syndrome were excluded from the healthy control and T2DM groups. The exclusion criteria for all participants were as follow: history and evidence of stroke, myocardial infarction, cancer, kidney disease, chronic inflammation, autoimmune diseases and the use of thiazolidinedione family drugs. The study was performed in accordance with the Declaration of Helsinki and approved by the ethical committee of AJA University of Medical Sciences. Written consent was obtained from all participants.

### Anthropometrics and biochemical measurements

A standard formula (weight (kg) / height (m^2^)) was used for the calculation of body mass index (BMI). Furthermore, a standard sphygmomanometer was used to measure the systolic blood pressure (SBP) and diastolic blood pressure (DBP) after 15 min of rest. After overnight fasting, venous blood was collected from all participants. Fasting blood glucose (FBG), triglycerides (TG), total cholesterol (TC), low density lipoprotein-cholesterol (LDL-C), high density lipoprotein-cholesterol (HDL), alanine aminotransferase (ALT), aspartate aminotransferase (AST) and creatinine (Cr) were measured using commercially available kits (Pars Azmoon, Iran). Also, the ELISA technique was used for measuring fasting insulin level (Monobind, USA). The homeostatic model assessment of insulin resistance (HOMA-IR) was calculated using the following equation: [FBG (mg/dL)] × [fasting blood insulin (μU/mL)] / 405 [20].

### Measuring cytokines and adipokines

Serum levels of IL-6, TNF-α and Metrnl were measured using commercial ELISA kits (R & D System, USA). Furthermore, the intra- and inter-assay coefficients of variation (CV) of IL-6 and TNF-α were 6.4 and 9.1, and 5.4 and 7.2, respectively. The intra and inter-assay CV for Metrnl measurement were 6.2 and 7.1, respectively. Also, the serum level of adiponectin was assessed by the ELISA technique (Adipogen, South Korea), with intra- and inter-assay CV of 3.4 and 4.3%, respectively.

### Statistical analysis

Categorical data are shown in frequency and percentage and tested by the Chi-square test. Continuous variables are given with mean and standard deviation (SD). The normality of continuous data was tested using the Kolmogorov-Smirnov test. Furthermore, normal distributed data was analyzed by one-way ANOVA with the Bonferroni post hoc test. Non-normal distributed data were evaluated by the Kruskal Wallis test supplemented by the Bonferroni test. In addition, the Spearman correlation test was used to evaluate the correlation between Metrnl as well as anthropometric and metabolic profiles. Non-normal distributed data were logarithmically transformed before correlation analysis. Analysis of covariance (ANCOVA) was carried out to remove the possible effects of covariates on the serum levels of Metrnl. Moreover, the association of Metrnl with the risk of T2DM and CAD presence were tested by multinomial logistic regression. All analyses were performed by SPSS version 16 (SPSS, USA) and a p value < 0.05 was considered as a significant threshold.

## Results

### Anthropometrics and biochemical measurements

Table 1 presents the anthropometric results, metabolic profiles and medications administered. There were no significant differences in terms of age (p = 0.152), sex (p = 0.095) and BMI (p = 0.280) between the studied groups. However, SBP was higher in the CAD group compared to the control. Also, the results showed significantly increased FBG and HOMA-IR in the T2DM group compared to the control and CAD groups. Furthermore, insulin was higher in the T2DM group compared to the control and CAD groups, and CAD compared to the control group. Moreover, TG was higher and HDL-C was lower in the T2DM group compared to the control group, while TC and LDL-C showed no significant differences between both groups.

**Table 1:** Anthropometric, metabolic and medications use of the studied population.

Variables		Control	CAD	T2DM	P Value
Age (year)		56.4 ± 7.5	59.4 ± 7.9	58.6 ± 7.8	0.154
Sex (male) [n (%)]		29 (70.7)	54 (81.8)	41 (65.1)	0.095
BMI (kg/m^2^)		26.0 ± 3.5	27.2 ± 4.1	26.3 ± 4.3	0.280
SBP (mmHg)		124.9 ± 13.1	137.2 ± 17.4^a^^**^	136.1 ± 20.0^b^^**^	0.001
DBP (mmHg)		77.4 ± 9.9	86.0 ± 13.3^a^^**^	83.7 ± 14.7	0.005
FBG (mg/dL)		95.8 ± 12.7	95.4 ± 11.7	160.3 ± 24.6^b^^**^^,^^c^^**^	< 0.001
Insulin (uU/ml)		4.0 ± 2.1	6.5 ± 4.7^a^^**^	10.4 ± 4.3 ^b^^**^^,^^c^^**^	< 0.001
HOMA-IR		0.95 ± 0.56	1.52 ± 1.10	4.19 ± 1.98 ^b^^**^^,^^c^^**^	< 0.001
TG (mg/dL)		126.6 ± 53.1	136.2 ± 49.5	154.7 ± 49.0 ^b^^*^	0.015
TC (mg/dL)		175.1 ± 40.6	172.9 ± 45.0	185.2 ± 43.8	0.246
LDL-C (mg/dL)		106.2 ± 33.4	104.6 ± 34.5	112.1 ± 36.9	0.462
HDL-C (mg/dL)		46.2 ± 6.4	43.3 ± 11.2	41.8 ± 4.9^b^^*^	0.029
Creatinine (mg/dL)		1.14 ± 0.14	1.15 ± 0.19	1.16 ± 0.15	0.774
AST (U/L)		18.3 ± 5.5	19.8 ± 6.8	19.3 ± 6.4	0.516
ALT (U/L)		18.7 ± 7.7	21.6 ± 8.7	19.0 ± 7.9	0.111
Smoker [n (%)]		10 (24.4)	27 (40.9)	24 (38.1)	0.201
Medication [n (%)]	Insulin +OHA^#^	0 (0)	0 (0)	28 (44.4)	< 0.001
	Statins	0 (0)	49 (74.2)	31 (49.2)	< 0.001

Continuous data are given in mean ± SD and categorical data are shown in frequency and percentage.

# OHA, oral hypoglycemic agents

* P value < 0.05

** P value < 0.01

a. Comparison between controls and CAD

b. Comparison between controls and T2DM

c. Comparison between CAD and T2DM

### Serum levels of cytokines and adipokines

The serum levels of IL-6 were significantly elevated in the CAD (8.31 ± 3.56 pg/mL) and T2DM (8.96 ± 3.33 pg/mL) groups compared to the control group (5.37 ± 1.8 pg/mL) (p < 0.001 for both). Likewise, the serum levels of TNF-α were found to be higher in CAD (27.6 ± 6.3 pg/mL) and T2DM (27.7 ± 8.0 pg/mL) patients compared to the control (22.3 ± 7.4 pg/mL) (p = 0.001 for both). While adiponectin was significantly lower in the CAD (8.4 ± 3.2 μg/mL) and T2DM (9.6 ± 2.6 μg/mL) groups compared to the control (11.2 ± 3.4 μg/mL) (p < 0.001 and p = 0.033, respectively) (Table 2).

**Table 2:** Serum levels of adipokine and inflammatory cytokines.

Variables	Control	CAD	T2DM	P Value
IL-6 (pg/mL)	5.37 ± 1.8	8.31 ± 3.56^a^^**^	8.96 ± 3.33^b^^**^	< 0.001
TNF-α (pg/mL)	22.3 ± 7.4	27.6 ± 6.3^a^^**^	27.7 ± 8.0^b^^**^	< 0.001
Adiponectin (μg/mL)	11.2 ± 3.4	8.4 ± 3.2^a^^**^	9.6 ± 2.6^b^^*^	< 0.001
Metrnl (pg/mL)	95.33 ± 32.56	75.18 ± 28.48^a^^**^	73.89 ± 33.60^b^^b^^**^	< 0.001

* P Value < 0.05

** P Value < 0.01

a. Comparison between controls and CAD

b. Comparison between controls and T2DM

The serum levels of Metrnl were found to be lower in CAD (75.18 ± 28.48 pg/mL) and T2DM patients (73.89 ± 33.60 pg/mL) compared to the control (95.33 ± 32.56 pg/mL) (p < 0.005, p < 0.003, respectively) (Table 2). Statistical analysis using ANCOVA test demonstrated that decreased serum levels of Metrnl in CAD (p = 0.046) and T2DM (p = 0.004) patients were independent from the covariates (age, sex, BMI and medications). Moreover, Metrnl showed no significant difference in males compared to females.

### Association of Metrnl with CAD and T2DM

**Fig 1:**
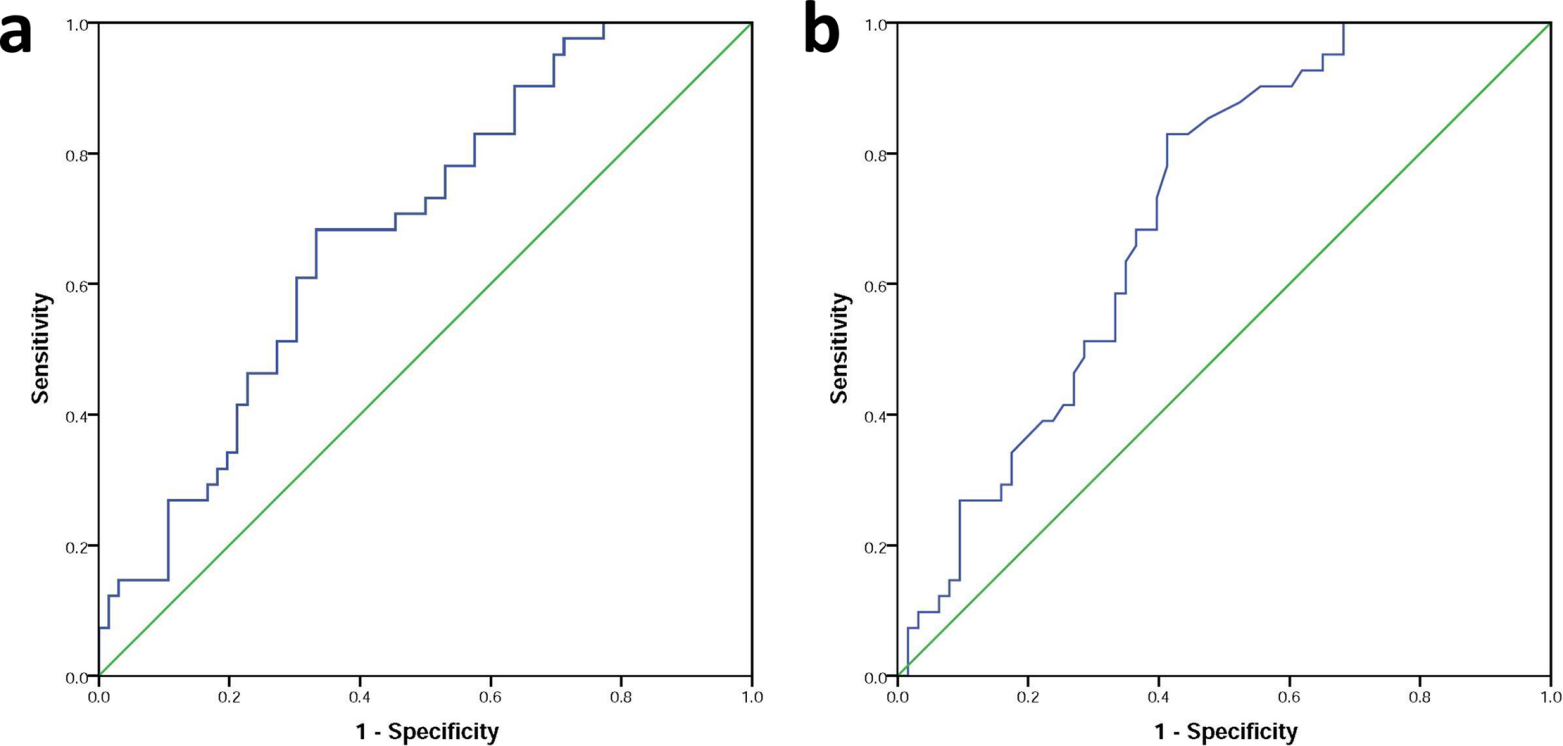
ROC curve for discriminating diseases status from control. a) ROC curve for discriminating CAD from control. b) ROC curve for discriminating T2DM from control.

Multinomial logistic regression revealed that decreased serum levels of Metrnl were associated with increased risks of CAD (OR [CI] = 0.982 [0.970–0.994]) and T2DM (OR [CI] = 0.981 [0.968–0.993]) incidence. Also, after adjusting for age, sex and BMI, the association with CAD and T2DM remained significant (Table 3). ROC curve analysis showed that a cut of the value of Metrnl = 74.86 pg/mL, had a relatively good sensitivity (68.3%) and specificity (66.7%) for discriminating CAD from the control (area under curve (CI): 0.684 (0.584–0.785), p = 0.001, Fig 1A). Also, discriminating T2DM from the control with a cut off value of Metrnl = 73.51 pg/mL had a relatively good sensitivity (68.3%) and specificity (63.5%) (area under curve (CI): 0.709 (0.612–0.807), p < 0.001, Fig 1B).

**Table 3:** Odds ratios of CAD and T2DM presence according to Metrnl serum levels.

Model	Group	Odds ratio (CI)	P Value
Crude models	CAD	0.982 (0.970–0.994)	0.004
	T2DM	0.981 (0.968–0.993)	0.002
Adjusted models*	CAD	0.980 (0.967–0.993)	0.004
	T2DM	0.981 (0.968–0.994)	0.004

*Adjustment carried out for age, sex and BMI.

### Correlation of Metrnl with anthropometric and biochemical parameters

Correlation analysis was performed in the control group and the results revealed that Metrnl significantly correlated with BMI (r = -0.318, p = 0.043) and HOMA-IR (r = -0.329, p = 0.036, Table 4).

**Table 4:** Spearman correlation of Metrnl with anthropometric and metabolic profiles.

	Control	CAD	T2DM
Age	-0.129	0.225	0.121
BMI	-0.318*	-0.146	-0.354**
SBP	-0.168	0.035	0.039
DBP	-0.077	0.034	0.075
FBG	-0.266	-0.192	-0.420**
Insulin	-0.308	-0.106	-0.228
HOMA-IR	-0.329*	-0.140	-0.338**
TG	-0.180	-0.111	-0.023
TC	-0.090	-0.013	-0.051
LDL-C	-0.070	0.087	-0.070
HDL-C	0.135	-0.227	-0.199
Adiponectin	-0.091	0.258*	0.174
TNF-α	-0.306	-0.294*	-0.335**
IL-6	-0.260	-0.319**	-0.296*
Creatinine	-0.222	-0.110	-0.055
AST	-0.203	-0.048	0.029
ALT	-0.132	0.032	-0.095

* P Value < 0.05

** P Value < 0.01

In addition, there was a positive correlation between Metrnl and adiponectin (r = 0.258, p = 0.036), in the correlation analysis of patients with CAD. Furthermore, Metrnl revealed a negative correlation with IL-6 (r = -0.319, p = 0.009) and TNF-α (r = -0.294,p = 0.017) (Table 4).

Metrnl demonstrated a negative correlation with BMI (r = -0.354, p = 0.004), FBG (r = -0.420, p = 0.001), HOMA-IR (r = -0.338, p = 0.007), TNF-α (r = -0.296) and IL-6 (r = -0.296, p = 0.018) in patients with T2DM (Table 4).

In addition, all significant correlations were adjusted for age and sex. All correlations remained significant after adjustment, except the correlation between Metrnl and HOMA-IR in the controls and correlation between Metrnl and adiponectin in CAD patients (S1 Table).

## Discussion

The relationship between adipose tissue and cardiometabolic disease is a matter of ongoing research [21]. Adipokines as secretory factors from the adipose tissue are considered as potential candidates for the relation of the adipose tissue with whole body glucose and lipids metabolism[1,9]. Studies have shown the perturbation of circulatory adipokines in the context of cardiometabolic diseases such as T2DM, metabolic syndrome, CAD and nonalcoholic fatty liver disease [22–26]. Metrnl is a newly discovered adipokine with favorable effects on insulin resistance [10,11,14]. In the present study, the serum levels of Metrnl decreased in T2DM and CAD patients compared to the controls. In contrast with the present study, Chung et al., showed that the circulating levels of Metrnl were increased in patients with T2DM[15]. This contradiction might be as a result of medication or ethnic differences. Furthermore, in Chung et al.'s study, there is no data on medications whereas the serum levels of lipids (TC, LDL-C and TG) significantly reduced in patients with diabetes. This result suggested intensive lipid lowering therapies in patients with diabetes in Chung et al.’s study. It has been shown that PPAR-γ downregulation attenuates improving insulin sensitivity by Metrnl; therefore, medication that interferes with PPAR-γ signaling might affect the circulating levels of Metrnl. Our study population was free from PPAR-γ antagonists. In addition, Chung et al., selected the study population from the Korean sarcopenic obesity study that included patients with sarcopenia [27], and it has been shown that Metrnl is secreted by skeletal muscle in humans and mice [13]. Therefore, sarcopenia could be a confounding factor in Chung et al.’s study that affects the circulating levels of Metrnl. In line with the present study, Lee et al. demonstrated lower levels of Metrnl in newly diagnosed T2DM patients [16]. In the present study, the results revealed that the serum levels of Metrnl decreased in T2DM and CAD patients independent of age, sex, BMI and medications. Furthermore, our results showed that the decreased serum levels of Metrnl had independent association with increased risk of T2DM and CAD. The exact mechanism for linking Metrnl to the pathogenesis of CAD and T2DM is yet to be clarified. However, experimental studies have reported several favorable actions of Metrnl, such as adipose tissue browning [13], inhabitation of inflammation [13,14], improving insulin sensitivity and improving adipocyte functions[14,23]. It has been shown that a deficiency of Metrnl in adipocytes promotes insulin resistance [14] whereas the upregulation of Metrnl in adipocytes improves insulin sensitivity [14]. Moreover, Metrnl promotes insulin induced AKT phosphorylation [14], and it has been shown that the favorable effects of Metrnl on insulin sensitivity are mediated by PPAR-γ[14]. In the present study, Metrnl was found to be correlated with insulin resistance and the parameter of glucose and insulin metabolism in T2DM patients. Also, Metrnl showed a negative correlation with HOMA-IR in the control, but this correlation disappeared after adjusting for age and sex. Previous studies have shown a negative correlation of Metrnl with the parameter of insulin resistance in the population with and without T2DM and obese patients [16,28], but our results showed that the relation of Metrnl with insulin resistance is significant in the context of T2DM. This result is further *in vivo* evidence for the relationship between Metrnl and insulin resistance.

Furthermore, Metrnl inhibits TNF-α expression in high fat diet induced inflammation [14]. Metrnl also increased anti-inflammatory cytokines in mice [13]. In the present study, Metrnl had negative correlations with IL-6 and TNF-α in CAD and T2DM patients. These results suggest the possible role played by Metrnl in inflammatory milieu in the context of CAD and T2DM. Based on the fact that both T2DM and CAD are pro-inflammatory based diseases and the serum levels of IL-6 and TNF-α are elevated in the diseases[17], thus it appears that inflammation plays key roles in either the induction or stimulation of the disorders. As a result of the anti-inflammatory effects of Metrnl, via downregulation of IL-6 and TNF-α, it may be hypothesized that Metrnl not only plays crucial roles in the regulation of insulin pathways, it also improves CAD and T2DM pathogenesis via the down-regulation of inflammation. However, according to the fact that cytokines play important roles in network manners, thus, it appears that more investigations by evaluation of other inflammatory and anti-inflammatory cytokines elucidate the roles of Metrnl on the immune system.

White adipose tissue is the main secretion organ for Metrnl [10]. Previous studies showed that Metrnl increased in obese mice [13,14], whereas in the present study a negative correlation was found between BMI and Metrnl in the controls and T2DM patients. This contradiction might result from differences in human and animal studies. In line with our results, a study has shown lower levels of Metrnl in obese subjects and increased levels of Metrnl after bariatric surgery [28].

The results of the present study showed independent association of Metrnl with CAD and T2DM presence, and negative correlation with insulin resistance and inflammation. These results suggest a possible role for Metrnl in the pathogenic mechanisms of CAD and T2DM, which could be a possible explanation for the association of Metrnl with CAD and T2DM. However, the cross sectional design of the present study limited us to conclude that there is a casual relationship between Metrnl and diseases.

## Supporting information

S1 Table Adjusted correlation of Metrnl with anthropometric and metabolic profiles. (DOCX)
