# Supplementary material for: Lower serum levels of Meteorin-like/Subfatin in patients with coronary artery disease and type 2 diabetes mellitus are negatively associated with insulin resistance and inflammatory cytokines
Source: PLoS One. 2018 Sep 13;13(9):e0204180. doi: 10.1371/journal.pone.0204180 (PMC6136801; doi:10.1371/journal.pone.0204180)
Supplement: S1 Table — (DOCX) [file pone.0204180.s001.docx]

S1 Table. Adjusted correlation of Metrnl with anthropometric and metabolic profiles.

|  | Control | CAD | T2DM |
| --- | --- | --- | --- |
| BMI | -0.323* | - | -0.347** |
| FBG | - | - | -0.419* |
| HOMA-IR | -0.267 | - | -0.355** |
| Adiponectin | - | 0.225 | - |
| TNF-α | - | -0.305* | -0.326* |
| IL-6 | - | -0.314* | -0.280* |

* P Value < 0.05

** P Value < 0.01
